# Two Experiments on the Psychological and Physiological Effects of Touching-Effect of Touching on the HPA Axis-Related Parts of the Body on Both Healthy and Traumatized Experiment Participants

**DOI:** 10.3390/bs8100095

**Published:** 2018-10-17

**Authors:** Chigusa Theresa Yachi, Taichi Hitomi, Hajime Yamaguchi

**Affiliations:** 1 Graduate School of International Humanities and Social Science, J. F. Oberlin University, 3758 Tokiwa-machi, Machida-shi, Tokyo 194-0294, Japan; y-hajime@obirin.ac.jp; 2 Graduate School of International Humanities and Social Science, J. F. Oberlin University, Faculty of Health Science Technology, Bunkyo Gakuin University, 1196 Kamekubo, Fujimino-shi, Saitama 356-8533; t-hitomi@bgu.ac.jp

**Keywords:** touching, RSA, HPA axis, ACE, PTSD, Somatic Experiencing™

## Abstract

Two experiments were conducted to measure both the psychological and physiological effects of touching on the HPA axis related parts of the body. HPA stands for the hypothalamus, pituitary, and adrenal. One experiment was conducted with a group of healthy experiment participants, and another was with a group of traumatized participants who had Adverse Childhood Experiences (ACE). In the experiments, the back of an experiment participant was touched, where a kidney-adrenal was supposed to reside, and both the psychological and physiological effects were measured. As a result, respiratory sinus arrhythmia (RSA), an indicator of the parasympathetic nerve system function and, especially, an indicator of the social engagement system increased, by a statistically significant degree, as a consequence of HPA touching in both the healthy and the trauma group, in comparison with the control. The traumatized participants had a lower RSA, and this was increased by HPA touching, accompanied by a decrease of the heart rate. It is worth noting that the social engagement function was possibly enhanced by HPA touching, especially in the trauma group, whose members tend to have difficulty being pro-social. This touching method is very simple, so it can be administered not only by oneself, but also by psycho-therapists and body workers in order to enhance both psychological and physiological well-being.

## 1. Introduction

### 1.1. Touching and Preceding Studies

This research was designed to examine the psychological and physiological effects of touching. This research involved two groups of experiment participants. One contained healthy people and the other consisted of those who had traumatic experiences and had gone through trauma healing treatment including touching therapy. This research examined if touching could support people in regaining their mental health, focusing on those who had ACE (adverse childhood experiences) and suffered from mild to clinical depression.

In the field of science, touching has not been widely studied. The psychological and physiological effects of touching have not been explored in the past. Only recently, it has received attention, and some scientific studies were conducted. It was reported, in these preceding studies, that touching had various effects, including the reduction of anxiety, stress, and pain; the enhancement of relaxation and the immune system; and the quickening of the healing of wounds [1,2,3,4,5,6]. Miyazaki et al. [7] reported that by touching the meridian or acupuncture points of adult experiment participants, their subjective freedom of neck movement was improved, and stress level was reduced. It became clear to Miyazaki et al. [7] that touching had a positive effect on the somatic sensation and psychological index. Furthermore, Mitsumori and Yamaguchi [8] reported that mothers who experienced baby massage had lower anxiety about child raising and showed a lower risk of child abuse, compared with mothers who did not.

Hasuo et al. [9] reported that the digestion speed of cancer patients with gastric fistula was enhanced by holding the hand of one of their family members. Hasuo et al. [9] found that digestion was enhanced by various factors, including not only hand-holding, but also the loving presence and eye contact of family members. The study showed the possibility that hand holding could positively affect the general visceral function of cancer patients. Furthermore, Hasuo et al. [9] reported that the family members had a significant feeling of helplessness and powerlessness, as they could not do very much to help their family member with cancer. Hasuo et al. [9] reported that the self-efficacy of family members was enhanced by the knowledge that their effort of hand-holding had a positive effect on the patient’s condition. By observing the work by Hasuo et al. [9], it can be said that touching had a positive effect on the physical function of those who were touched and on the psychological state of those who touched.

Yamaguchi [10] discussed the mechanism of C tactile fibers and how they could positively affect the body and mind. C tactile fibers were most activated when the experiment participants were touched at a speed of 5 cm per second. It was known that their electric signals traveled at a relatively low speed and reached a deep and wide area of the brain. The electric signal could reach to the brain stem, which controls the basic survival functions, such as breathing and heart beating; the amygdala, which controls emotions; the hypothalamus, which controls the autonomic nervous system and hormones; and the insula, which controls affect.

Yamaguchi [10] further discussed the mechanism by which C tactile fibers could reduce stress. When a system was under stress, the reaction would occur in the sequence of “hypothalamus → pituitary → adrenal”. Then, cortisol would be emitted from the adrenal and move through the body, negatively affecting viscera. According to Yamaguchi [10], tactile fibers would block the electric signals that travel to the brain, so that less pain or suffering would be experienced, even under stress or injury. The preceding study shows that touching could influence the stress response, which was controlled by the hypothalamus, pituitary, and adrenal (HPA) axis. 

### 1.2. Touching and the HPA Axis

The HPA axis is a triangle connecting the hypothalamus, pituitary, and an adrenal of both sides of the back of the body. This triangle is known to control stress reaction. An amygdala is a part of the limbic system, which grasps danger, and signals are sent to the hypothalamus and pituitary, which emit neuro transmitters to the adrenals. Once the signals are caught by the adrenals, they will excrete adrenalin. If the stressful state continues over a certain limit, cortisol will be excreted to replace adrenalin. In case of danger, it is necessary to excrete the stress hormone to have appropriate survival reactions, but if such a stressful state continues and the excretion of the stress hormone continues for a long period of time, when that high level of sympathetic surge is no longer necessary, this may cause damage to organs, as well as physical and mental disease. The HPA axis is very closely related to the stress response. However, there are no preceding studies that examined the effect of touching on the HPA axis-related parts of the body.

### 1.3. Respiratory Sinus Arrhythmia (RSA) and Touching

This research focused on respiratory sinus arrhythmia (RSA) to measure the effect of touching. RSA is a natural fluctuation of the heart beat. When a person is relaxed, the heart rate increases slightly during inhalation, and decreases slightly during exhalation. It is a natural response of the heart to do less work when it is safe in order to reduce its workload, as it continues non-stop from the first to the last breath of a person. Porges [11] claimed that RSA was caused by the vagus nerves, and the level of RSA reflects the function of one’s social engagement system. The human ventral vagus nerve system originates in a nucleus ambiguus in the brain stem and primarily connects to the viscera through the respiratory diaphragm. A part of it is connected to the sino arterial node of one’s heart in order to control the heart rate. Therefore, it is called the “vagal break” and sometimes the “cardiac vagus nerve branch”.

This “vagal break” controls the heart rate without the intervention of the sympathetic nerve system. This is how RSA occurs. If this ventral vagus nerve system is functioning well, one can maintain social engagement when the body feels safe. Under stress, people normally go into a “fight/flight” mode led by the sympathetic nerve system. However, if the social engagement system is functioning well, it down-regulates the sympathetic response. Porges [11] argues that the phylogenetically newer system, in this case, the ventral vagus nerve system, down-regulates the phylogenetically older sympathetic nerve system.

RSA was found to be higher among men than women, and it became lower as people aged [12]. A preceding study reported that development of the nerve system of premature babies was enhanced when they received skin to skin touching from their mother. This was indicated by the increase of RSA [13]. However, even if the premature babies received touching and their RSA was increased, their RSA still fell behind that of the babies born full term [14]. RSA is measured by an electrocardiogram in a non-invasive way.

### 1.4. RSA and Post Traumatic Stress Disorder (PTSD)

There are several preceding studies concerning the relations between RSA and PTSD. If mammals undergo stress, their sympathetic nerve system surges. However, if their ventral vagus nerve system was robust, it was possible to raise the heart rate without the intervention of the sympathetic nerve system [15]. People with PTSD generally had a lower RSA, and their lower RSA became even lower as a consequence of stress. Their heart rate increased under the influence of the sympathetic nerve system [16,17].

Sahar, Shalev, and Proges [18] reported that RSA increased under stress in the non-PTSD group. RSA decreased under stress in the PTSD group, with no changes in the physiological data. In the PTSD group, under stress, the RSA level decreased, and the heart rate increased. Porges [13] and Patriqui, Scapa, Friedman, and Porges [19] claimed that the PTSD group recognized stress and interpreted that there was a danger. They tended to misperceive the danger to be life threatening. Then, their sympathetic nerve system became stimulated. Furthermore, Patriqui, et al. [19] claimed that people with PTSD had a poorly functioning social engagement system. This was because the function of their ventral vagus nerve system was compromised as a result of PTSD. Therefore, they had poor facial expressions as well as poor neck and head turning functions. This matched with the finding that their RSA was lower at the base line as well as under stress. RSA reflected the function of the social engagement system [19].

### 1.5. Adverse Childhood Experiences (ACE)

The adverse childhood experiences (ACE) study was first conducted jointly by CDC (the Center for Disease Control) and the Kyser Permanente Hospital from 1995 to 1997 in South California [20]. Approximately 17,000 patients, who were treated at the Kyser Permanente Hospital, joined the study. The ACE study has continued until now, and various follow-up studies have been conducted. The first study was conducted primarily on a Caucasian population with middle-class income. All of the participants were covered by insurance. As a result, it was found that one out of three had at least one ACE score, and one out of five had multiple ACE scores. If a study participant had more ACE scores, his/her health and quality of life (QOL) were more challenged and compromised.

**Figure 1:**
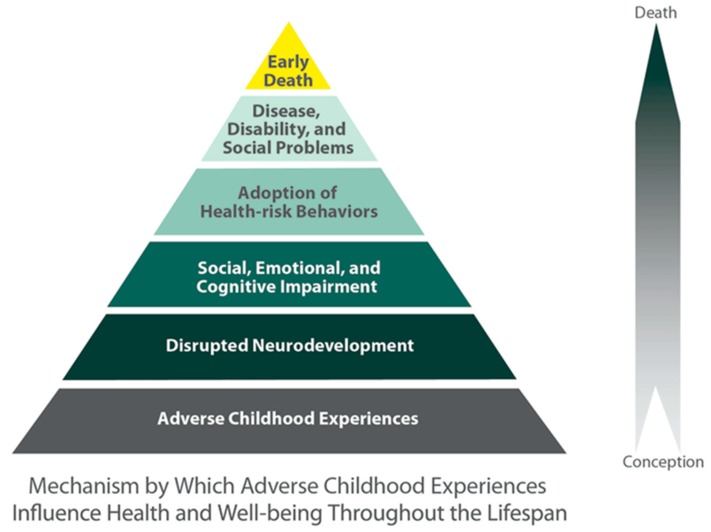
Adverse childhood experiences (ACE) pyramid.

The negative consequences of ACE included an increased risk of disease and lower QOL. People with a high ACE score had a higher likelihood of developing alcohol and drug abuse, smoking, depression, ischemic heart disease, chronic lung disease, liver disease, sexually transmitted disease, and so on. People with a high ACE score had a higher likelihood of starting smoking, drinking, abusing drugs, having sexual intercourse, unwanted pregnancy, multiple sexual partners, being sexually victimized, experiencing the death of an embryo, suffering violence from an intimate partner, having poor performance at school and in the workplace, living in poverty, making suicide attempts, and committing suicide in the early stage of their lives. The following is the so-called ACE pyramid, which depicts the health effect of ACE from conception to the death (Figure 1). People with ACE tended to have various symptoms, which were considered to be PTSD [21].

In relation to RSA, women with ACE had a lower RSA, and it took them longer to recover after stress [22]. Women with ACE had less cortisol excretion after a stress test, indicating a possible change in their hormone excretion system [23]. The preceding studies showed that ACE negatively affected the development of the brain, altered the endocrine system, and lowered QOL, and there was a difference in these effects between males and females [18,24,25].

## 2. Materials and Methods

Experiment 1. Healthy group.

### 2.1. Experiment Participants

Some preceding studies reported that ACE could alter physiology [20,26,27]. It was also made clear that smoking and drinking affected physiology [28]. Therefore, in this experiment, only women with a low or no ACE score, who were non-smoking/minimum smoking, consumed a moderate amount or no alcohol habitually, and did not take any medication were chosen.

As participants, 13 healthy young women were selected. The healthy group included 11 college and graduate school students and 2 working women. Their age was between 20 and 32, with an average age of 24.23 ± 3.91, average ACE score of 0.69 ± 0.75, and average Davidson Trauma Scale (DTS) score of 20.46 ± 14.68.

### 2.2. The Therapist

The therapist was a 57-year-old woman and a certified Somatic Experiencing™ practitioner.

### 2.3. Ethical Consideration

Both written and verbal explanation was given before the experiment, so that participation in the experiment was voluntary and one would not be penalized for not participating or withdrawing from the experiment. The college students were instructed to go to the “college counseling room” if they felt that they would need to share some of the feelings or thoughts caused by the experiment. This experiment was approved by the J. F. Oberlin University Research Ethics Committee (Approval number 16049).

### 2.4. Items Examined

The participants filled out both the ACE (adverse childhood experiences) [21] and DTS (Davidson Trauma Scale) questionnaires at the beginning of the experiment [29].

**Figure 2:**
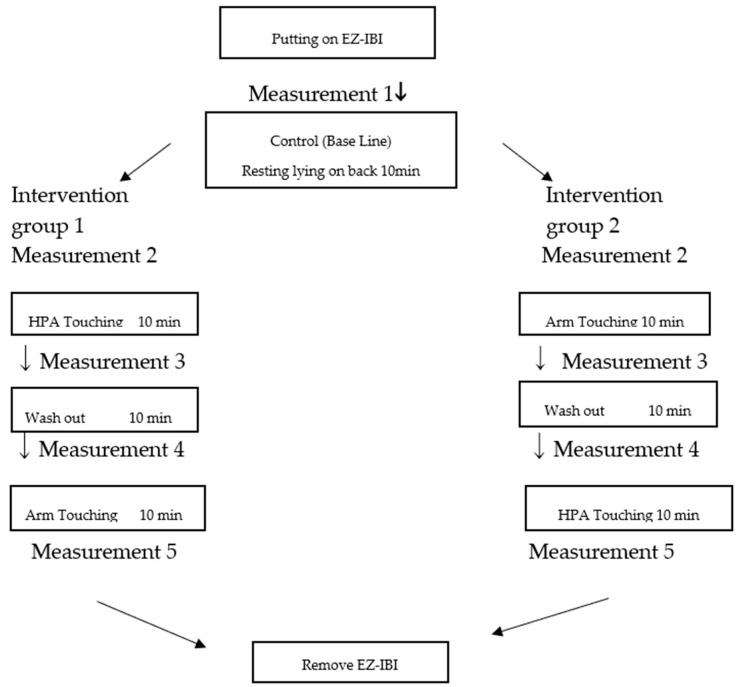
Flow of the experiment. HPA—hypothalamus, pituitary, and adrenal.

Measurements were conducted five times in one experiment, as depicted in the graphic of the flow of the experiment (Figure 2). 

In each measurement, Two Dimensions Mood Scale test (TDMS-t) [30], heart rate, blood pressure, saliva amylase concentration, and RSA were measured. The equipment used for heart rate and blood pressure measurement was EW-BW13 portable blood pressure meter by Panasonic (Monma, Osaka Prefecture, Japan). For amylase concentration, a Nipro Amylase Monitor (Osaka City, Osaka Prefecture, Japan) was used. For RSA, EZ-IBI cardiogram by UFI company (Morro Bay, CA, USA) was used. Then, the data were processed by Cardio Edit/Cardio Batch software developed by Porges (Bloomington, IN, USA) [11].

### 2.5. Method

The following three conditions were provided.

1. Base line: Resting on a massage table, lying on her back for 10 min.
2. Arm touching: A participant lay on a massage table, on her back, and the therapist put her right hand on the upper arm of the participant. The participant was touched for 5 min on the right, and for another 5 min on the left, with a total of 10 min.
3. HPA touching: A participant lay on a massage table, on her back, and the therapist slid her right hand under the back of the participant. The participant was touched for 5 min on the right, and for another 5 min on the left, with a total of 10 min.

To eliminate the effect of order, half of the participants received HPA touching first, then arm touching. The other half received the arm touching first, and then HPA touching.

The experiment room was a class room of the J. F. Oberlin University. The room temperature was kept at 26 °C, and nobody was allowed in the room during the experiment.

## 3. Experiment 2 Trauma Group

### 3.1. Experiment Participants

As participants, 11 female clients, who were receiving Somatic Experiencing™ sessions at the Institute, were solicited. The participants’ average age was 49.71 ± 10.47, with an average ACE score of 4.00 ± 1.84 and average DTS score of 50.90 ± 26.74.

The trauma group experiment participants were the clients of the therapist. They knew each other, and there was already a trusting relationship between the participants and the therapist. There was a concern that this fact might interfere with the experiment result. However, touching would usually be done between people who know each other and have pre-existing relationships. The therapeutic touching would seldom be done between two complete strangers. Furthermore, the preceding studies reported that the stress response decreased when the experiment participants were touched by someone who they trusted, and the stress response increased when they were touched by someone who they did not know [31,32]. As the therapeutic touching was expected to occur between two people with a pre-existing relationship, it was considered worthwhile to conduct the experiment with a therapist and clients with pre-existing relationships.

It has to be noted that the trauma group experiment participants were the clients of the therapists and had experienced HPA touch therapy prior to the experiment. They received 5 to 15 sessions, which partly involved kidney touching. The sessions lasted about 50 min and were administered mostly once a month.

### 3.2. The Therapist

The same as the Experiment 1.

### 3.3. Ethical Consideration

The same as the Experiment 1.

### 3.4. Items Examined

The same as the Experiment 1.

### 3.5. Method

The same as the Experiment 1, except the room. The counseling office of the therapist at the Institute was used and the other conditions were the same as those of the Experiment 1.

### 3.6. Flow of the Experiment

The same as the Experiment 1.

## 4. Statistical Analysis

The comparison was made within the participants. The correlation between age, ACE, and DTS was examined by *t*-test. The data of TDMS-t, heart rate, blood pressure, amylase concentration, and RSA were processed first to see if they followed the normal distribution. Once the normal distribution was observed, the data were then processed by analysis of variance (ANOVA). As the effect was found, multiple comparison was done by the Holm method. The statistical software used was HAD [33].

The sample size was small and there was some concern about type 1 error using ANOVA. Therefore, MANOVA was considered to be applied in addition to ANOVA using SPSS (IBM Japan, Chuoku, Tokyo, Japan). The spherical assumption was tested and Greenhouse–Geiser was 0.09. Greenhouse–Geiser was under 0.75, therefore, MANOVA was not conducted.

## 5. Results

### 5.1. t-Test of ACE, DTS, and RSA

ACE, DTS, and RSA of the healthy group and the trauma group were compared using the *t*-test. Statistical significance was obtained with ACE, DTS, and RSA (*p* = 0.00 **). Table 1 shows the result of the *t*-test of adverse childhood experiences (ACE), Davidson Trauma Scale (DTS), and respiratory sinus arrhythmia (RSA) of the healthy group and the trauma group.

As the result, there was statistical significance of *p* = 0.00 ** in ACE, DTS, and RSA of the healthy and the trauma group. ACE and DTS of the trauma group was significantly higher than those of the healthy group, and RSA of the trauma group was significantly lower than that of the healthy group.

### 5.2. Correlation of Age, ACE, DTS, and RSA

The correlations between age, ACE, DTS, and RSA were examined by correlational analysis. Age and ACE had strong correlation (r = 0.56) (*p* < 0.01 **). Age and DTS had correlation (r = 0.49) (*p* < 0.05 *). Age and RSA had strong correlation (r = −0.62) (*p* < 0.01 **). RSA and ACE had weak correlation (r = −0.35) (*p* < 0.10 ^+^). DTS and ACE had weak correlation (r = 0.40) (*p* < 0.10 ^+^). There was no correlation between RSA and DTS. Table 2 shows the result of the correlation analysis of age, ACE, DTS, and RSA of the healthy group and the trauma group.

### 5.3. The Result of ANOVA of S Value of TDMS-t

The Two Dimension Mood Scale test (TDMS-t) had four indices: V = Vitality, S = Stability, P = Positivity, and A = Arousal. TDMS-t indicated that among the healthy group, S value increased at the base line (*p* = 0.00 **). Among the trauma group, S value increased at the base line, and the main effect was observed (*p* = 0.00 *). Overall, the experiment participants showed decrease of tension and increase of relaxation during resting and touching, but statistical significance was not observed between pre- and post-touching. Table 3 shows the result of multiple comparison of S value of the healthy group by touching conditions. Table 4. Shows the result of multiple comparison of S value in the trauma group.

S value increased at the base line in the healthy group (*p* = 0.00 **). Then, the multiple comparison was conducted by the Holm method.

As the result of multiple comparison of S value among the trauma group, the main effect was obtained (main effect *p* = 0.04 *).

### 5.4. Blood Pressure

Blood pressure decreased during resting and touching, but statistical significance was not observed.

### 5.5. Heart Rate

The heart rate decreased after HPA touching compared with that in the base line (*p* = 0.01 *) among the trauma group. However, the main effect was not observed. Table 5 Shows the result of ANOVA of heart rate of the trauma group. Table 6 shows the result of multiple comparison of heart rate of the trauma group.

Heart rate decreased with statistical significance in consequence of HPA touching compared with the base line.

As the result of multiple comparison, no main effect was obtained.

### 5.6. Amylase Concentration

There were no significant changes in amylase concentration. Concerning amylase concentration, the result did not have normal distribution property. Therefore, ANOVA was not conducted.

### 5.7. RSA

Among the healthy group, RSA increased at statistically significant level with HPA touching (F (1,36) = 12, ηp^2^ = 0.277, *p* = 0.01 *, main effect *p* = 0.04 *). Among trauma group, RSA increased at statistically significant level with HPA touching (F (1,27) = 10, ηp^2^ = 0.210, *p* = 0.02 *, main effect *p* = ns). Table 7. show the result of ANOVA of RSA of the healthy group. Table 8 shows the result of the multiple comparison of RSA of the healthy group. Table 9 Shows the result of ANOVA of RSA of the trauma group. Table 10 shows the result of the multiple comparison of RSA of the trauma group.

As the result of ANOVA of RSA, the statistical significance of condition was obtained (*p* = 0.000 **). Then the multiple comparison was done by the Holm method.

RSA increased by both the Arm touching (*p* = 0.04 *) and the HPA touching (*p* = 0.01 *). No main effect was obtained either by the base line or the arm touching. The main effect was obtained by the HPA touching (main effect *p* = 0.04 *).

As the result of ANOVA of RSA of the trauma group, the statistical significance of the conditions was obtained (*p* = 0.02 *). Then, the multiple comparison was done by the Holm method.

RSA increased with statistical significance by HPA touching (*p* = 0.02 *). No statistical significance was obtained on the base line or on the arm touching. No main effect was obtained on the base line, the arm touching, or the HPA touching.

## 6. Discussion

### 6.1. Age, ACE, DTS, and RSA

There was strong correlation between age and ACE, DTS, and RSA. However, there was a noticeable difference of the average age between the healthy group and the trauma group; therefore, these results shall be considered as a reference. There was a negative correlation between ACE and RSA (r = −0.35) (*p* < 0.10 ^+^). It was speculated that when people had multiple ACE scores, their physiological system was negatively affected and their RSA became lower than that of the healthy group. This result matched with that of the preceding study [13]. There was no correlation between DTS and RSA. DTS measured the traumatic symptoms in a week prior to the experiment. This means that DTS was more about the recent traumatic symptoms. Even if the participants had higher DTS scores, their RSA was not affected. However, those with ACE had lower RSA. Again, it was possible that ACE affected the participants and altered their physiology.

It should be noted that RSA decreases in relation to age. In this experiment, the average age of trauma group is significantly higher than that of the healthy group. There is a possibility that RSA was lower among the trauma group partly because of their age. 

ACE and DTS had a weak correlation (r = 0.40) (*p* < 0.10 ^+^). It matched with the result of the preceding study reporting that people with ACE would have problems such as depression and anxiety even in their adulthood [29].

### 6.2. Psychological Index

In this research, psychological index was measured by TDMS-t [30]. The results of TDMS-t [30] did not show significant changes pre and post-interventions. Only the S-stability value increased with statistical significance in the base line for both the healthy and trauma group. It suggested that the experiment participants of both groups were relaxed as they were asked to lie down on the massage table. The therapist told the participants that the experiment required them simply to lie down and if they felt uncomfortable, they could express their feelings and the therapist would stop the experiment with no penalty. It seemed that the participants felt more stable at the first resting period at the base line, and the result indicated that they maintained the calm state during the experiment.

### 6.3. Physiological Index

The blood pressure showed no statistically significant change in either the healthy or the trauma group. As indicated by the psychological index, the participants were relaxed during the first resting period, and they maintained that calm state during the experiment. It can be said that, because the participants were relaxed during the experiment, their blood pressure did not show significant changes.

The heart rate did not show any statistically significant changes in the healthy group. In the trauma group, the heart rate dropped slightly with arm touching. A statistically significant decrease was observed with HPA touching, compared with the base line (*p* = 0.04 *).

The amylase concentration did not show any statistically significant changes. In this research, there was no stress cast on the participants. It could be speculated that significant change in the amylase concentration was not observed because of that fact that there was no stress cast during the experiment. Further study would be required to comment on the amylase concentration.

RSA, an indicator of the parasympathetic nerve system function and, especially, an indicator of the social engagement system, as discussed by Porges [13], increased to a statistically significant degree as a consequence of HPA touching in both the healthy and the trauma group. Traumatized participants had a lower RSA, which was increased by HPA touching, accompanied by a decrease of the heart rate. It was suggested that the ventral vagus nerve system of the participants with a history of trauma was activated by HPA touching. It was also suggested that, not only among healthy individuals, but also in traumatized individuals, the social engagement system, as indicated by RSA, was stimulated and increased by HPA touching.

In the future, I hope to conduct a comparison of different touching therapy modalities upon the trauma group. I am planning to conduct experiments featuring cranio sacral touch therapy. It is desired to study other touching modalities such as tapping touch, Though Field Therapy (TFT). I also hope to conduct a comparison of touching therapy and other forms of therapy upon the trauma group.

## 7. Conclusions

Average ACE and DTS scores were higher by a statistically significant degree, and the average RSA was lower by a statistically significant degree in the trauma group, compared with the healthy group. ACE score and RSA had a weak correlation. ACE and DTS scores had a weak correlation. In the healthy group, RSA increased by a statistically significant degree as a consequence of HPA touching (F (1,36) = 12, ηp^2^ = 0.277, *p* = 0.01 *, main effect *p* = 0.04 *). In trauma group as well, RSA increased by a statistically significant degree as a consequence of HPA touching (F (1,27) = 10, ηp^2^ = 0.210, *p* = 0.02 *, main effect *p* = ns). In the trauma group, the heart rate decreased as a consequence of HPA touching to a statistically significant degree (F (1,27) = 10, pη₂ = 0.386, *p* = 0.04 *). RSA, an indicator of the parasympathetic nerve system function, and possibly an indicator of the social engagement system, increased to a statistically significant degree as a consequence of HPA touching in both the healthy and the trauma group.

Touching on the HPA axis-related body parts was found to positively affect the social engagement system.

## 8. Limitations

### 8.1. Control of Participants/Age and Exposure to the Touching Therapy

In this research, the age of the participants in Experiment 1 was between 22 and 32, and that in Experiment 2 was between 32 and 69 years old. In the future, the age of participants will need to be better controlled. The trauma group experiment participants were the clients of the therapists and had experienced HPA touch therapy prior to the experiment. They received 5 to 15 sessions, which partly involved kidney touching. The touching session lasted about 50 min and was administered once a month. To eliminate the influence of the past therapy, trauma patients without exposure to HPA touch therapy shall be chosen in the future studies.

### 8.2. Place of Experiment

Experiment 1 was conducted in a university class room, and Experiment 2 was conducted in the therapist’s counseling room. The participants of Experiment 2 met the therapist on a regular basis, so it was very likely that the participants of Experiment 2 were more relaxed during the experiment, with a familiar therapist and a familiar place. In the future, the place of the experiments will need to be better controlled.

### 8.3. Psychological Scale

In this research, TDMS-t was used, but no significant change of psychological conditions was observed in the experiments. It will be necessary to add other types of psychological scales in the future.

### 8.4. Trauma History Questionnaire

In this research, the ACE [21] and DTS [29] questionnaires were used to gather trauma-related information. In addition to these, it will be necessary to introduce other trauma scales, so that trauma-related life events can be comprehensively listed.

## Figures and Tables

**Table 1:** The result of the *t*-test of adverse childhood experiences (ACE), Davidson trauma scale (DTS), and respiratory sinus arrhythmia (RSA) of the healthy group and the trauma group.

	Test	*t*	df	*p*	
ACE t-Test	Welch Test	5.57	12.80	0.00	**
	*t-Test*	5.93	22.00	0.00	**
DTS t-Test	Welch Test	3.37	14.94	0.00	**
	*t-Test*	3.53	22.00	0.00	**
RSA t-Test	Welch Test	3.46	21.14	0.00	**
	*t-Test*	3.46	22.00	0.00	**

** *p* < 0.01.

**Table 2:** The result of the correlation analysis of age, ACE, DTS, and RSA of the healthy group and the trauma group.

	Age		ACE		DTS	RSA
Age	1					
ACE	0.558	**	1			
DTS	0.486	*	0.402	^+^	1	
RSA	−0.62	**	−0.354	^+^	−0.164	1

** *p* < 0.01, * *p* <0.05, ^+^ *p* < 0.10.

**Table 3:** The result of multiple comparison of S value of the healthy group.

Multiple Comparison			Holm Method			
	df	*p*	Adjusted *p*		Main Effect *p*	
Base Line	10	0	0	**	0	**
Arm Touching	10	0.3	ns		0.3	ns
HPA Touching	10	0.2	ns		0.22	ns

** *p* < 0.01, ns: not significant. HPA-hypothalamus, pituitary, and adrenal.

**Table 4:** The result of multiple comparison of S value in the trauma group.

Multiple Comparison			Holm Method		
	df	*p*	Adjusted *p*	Main Effect *p*	
Base line	8	0.11	ns	0.04	*
Arm touching	8	0.54	ns	0.45	ns
HPA touching	8	0.58	ns	0.5	ns

* *p* < 0.05, ns: not significant.

**Table 5:** The result of ANOVA of heart rate of the trauma group.

Variables	df1	df2	*F*	pη2	*p*	
Pre-Post	2	20	6.29	0.39	0.01	*
Conditions	1	10	0.22	0.02	0.65	ns
Pre-Post * Conditions	2	20	0.86	0.08	0.44	ns

* *p* < 0.05, ns: not significant. ANOVA-analysis of variance.

**Table 6:** The result of multiple comparison of heart rate of the trauma group.

Multiple Comparison			Holm Method		
	df	*p*	Adjusted *p*	Main Effect *p*	
Base line	10	0.13	ns	0.19	ns
Arm touching	10	0.91	ns	0.93	ns
HPA touching	10	0.49	ns	0.56	ns

ns: not significant.

**Table 7:** The result of ANOVA of RSA of the healthy group.

Variables	df1	df2	*F*	pη2	*p*	
Pre-Post	2	24	1.35	0.1	0.28	ns
Conditions	1	12	15.35	0.56	0	**
Pre-post * Conditions	2	24	0.18	0.01	0.83	ns

** *p* < 0.01, * *p* < 0.05, ns: not significant.

**Table 8:** The result of the multiple comparison of RSA of the healthy group.

	df	*p*	Adjusted *p*		Main Effect *p*	
Base line	12	0.13	ns		0.23	ns
Arm touching	12	0.04	0.04	*	0.1	ns
HPA touching	12	0.01	0.01	*	0.04	*

* *p* < 0.05, ns: not significant.

**Table 9:** The result of ANOVA of RSA of the trauma group.

Variables	df1	df2	*F*	pη^2^	*p*	
Pre-Post	2	18	1.7	0.16	0.21	ns
Conditions	1	9	8.67	0.49	0.02	*
Pre-Post * Conditions	2	18	0.35	0.04	0.61	ns

* *p* < 0.05, ns: not significant.

**Table 10:** The result of the multiple comparison of RSA of the trauma group.

Multiple Comparison			Holm Method			
	df	*p*	Adjusted *p*		Main Effect *p*	
Base line	9	0.08	ns		0.27	ns
Arm touching	9	0.72	ns		0.84	ns
HPA touching	9	0.02	0.02	*	0.13	ns

* *p* < 0.05, ns: not significant.
